# Determining the efficacy of ExThera Seraph100 blood filtration in patients diagnosed with pancreatic cancer through the liquid biopsy

**DOI:** 10.1038/s44276-024-00069-3

**Published:** 2024-06-27

**Authors:** Stephanie N. Shishido, Divya Suresh, George Courcoubetis, Brandon Ye, Emmeline Lin, Jeremy Mason, Ken Park, Michael Lewis, Ruoxiang Wang, Simon K. Lo, Peter Kuhn, Stephen Pandol

**Affiliations:** 1 Convergent Science Institute for Cancer, Michelson Center, University of Southern California, Los Angeles, CA 90089 USA; 2 Institute of Urology, Catherine & Joseph Aresty Department of Urology, Keck School of Medicine, University of Southern California, Los Angeles, CA 90033 USA; 3 Norris Comprehensive Cancer Center, Keck School of Medicine, University of Southern California, Los Angeles, CA 90033 USA; 4 Pancreatic and Biliary Diseases Program, Cedars Sinai Medical Center, Los Angeles, CA 90048 USA; 5 Departments of Medicine and Pathology, Cedars Sinai Medical Center, Los Angeles, CA 90048 USA; 6 Department of Pathology, VA Greater Los Angeles Medical Center, Los Angeles, CA 90073 USA; 7 Center for Cancer Research and Development, Clark Atlanta University, Atlanta, GA 30314 USA; 8 Department of Biomedical Engineering, Viterbi School of Engineering, University of Southern California, Los Angeles, CA 90089 USA; 9 Department of Aerospace and Mechanical Engineering, Viterbi School of Engineering, University of Southern California, Los Angeles, CA 90089 USA; 10 Department of Biological Sciences, Dornsife College of Letters, Arts, and Sciences, University of Southern California, Los Angeles, CA 90089 USA

## Abstract

**Background:**

Cancer becomes lethal as it spreads from the primary site to the rest of the body. Circulating tumor cells (CTCs) are biomarkers of disease progression and have been associated with decreased overall survival. Blood filtration is a novel concept for removing CTCs from circulation to improve patient prognosis.

**Methods:**

This study utilizes liquid biopsy to assess the efficacy of ExThera Medical’s Seraph® 100 Microbind® Affinity Blood Filter on the blood of patients with pancreatic ductal adenocarcinoma (PDAC) using the third generation high-definition single cell assay workflow. Blood samples from treatment-naïve PDAC patients were collected and analyzed to characterize the CTCs and other rare cells present before and after filtration.

**Results:**

Examination of 6 paired portal vein blood (PoVB) samples demonstrated a statistically significant decrease in total rare cells, total cytokeratin (CK)+ cells, and CTCs across all patients due to filtration. Furthermore, analysis of 2 paired peripheral blood (PB) samples showed a decrease in total rare cells, total CK+ cells, and specific phenotypes of rare cells after filtration.

**Discussion:**

These preliminary results demonstrate initial proof of concept that this filtration device can remove CTCs from circulation and may therefore be useful as a therapy or adjunct in PDAC patient care.

## Introduction

Cancer becomes lethal when it spreads from the primary site to the rest of the body. Circulating tumor cells (CTCs) are considered a surrogate marker for metastatic activity and disease progression. The incidence of the CTCs has been associated with decreased overall survival [1], suggesting that decreasing CTC counts can improve patient prognosis. Blood filtration is a promising concept of removing cancer cells and vesicles [2] from the circulation which may interrupt the metastatic cascade and work as a therapeutic to improve patient outcomes. While current filtration methodologies based on apheresis are cited as fast, simple, and having high-throughput performance, many face issues such as clogging, decreased cell viability, and a drop in blood pressure which may pose harm to the patient [3]. Additionally, most filtration devices focus on enriching cells for further analysis, and do not return filtered blood to the patient [4, 5]. New filtration technologies are in development to treat cancer.

The liquid biopsy is one of the methods used to evaluate the efficacy of a filtration device in cancer care. Liquid biopsy is a technology that allows for real-time monitoring of disease progression [6] through analysis of CTC, as well as other tumor associated analytes such as oncosomes [7, 8]. Previous studies using liquid biopsy have correlated CTC incidence with tumor aggressiveness, metastasis, and decreased overall survival [9–11]. More recent studies from a study of curable (localized) patients support the correlation of CTC detection not only with survival but also to earlier onset of metastasis [12, 13]. In the study presented here, we utilize the liquid biopsy to assess the removal of CTCs by the the Seraph™ 100 Microbind™ Affinity Blood Filter (Seraph 100) from the blood of patients with pancreatic ductal adenocarcinoma (PDAC).

This study aims to understand the performance of the Seraph100 and its application to PDAC clinical care using the High-Definition Single Cell Assay (HDSCA3.0) workflow. This liquid biopsy method can detect and characterize epithelial, mesenchymal, endothelial, and hematopoietic cells, as well as oncosomes (large extracellular vesicles) [14–19]. We show the initial proof of concept that the Seraph100 can selectively remove CTCs from the blood of PDAC patients collected during endoscopic ultrasound fine needle aspiration (EUS-FNA).

## Materials and methods

### Study design

**Fig. 1:**
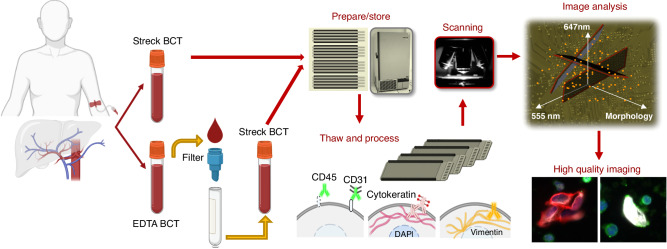
Study Overview and HDSCA3.0 Workflow. The PoVB and PB samples were filtered through Seraph100 mincolumns. After filtration, the cellular fraction was plated on slides and staining with the Landscape immunofluorescence assay. The slides were then scanned and analyzed using OCULAR rare event detection.

Portal vein blood (PoVB) from six treatment-naïve PDAC patients at Cedars Sinai Medical Center were taken during the EUS procedure. The small sample size is suitable for this pilot study in which the findings can provide enough data to perform preliminary statistical analyses, which can highlight trends and inform the design of larger, more definitive studies in the future. For two of the six patients, paired peripheral blood (PB) samples were taken prior to the procedure for comparison. Two tubes of blood per anatomical location were collected, with one tube processed through a sterile Seraph100 minicolumn (ExThera Medical Corporation, Martinez, CA). One original unfiltered sample and the matching filtered sample were shipped to Convergent Science Institute in Cancer (CSI-Cancer) overnight for analysis by HDSCA3.0 (Fig. 1). A total of 12 portal blood and 4 peripheral blood samples were collected between July 2022 and August 2022. Demographic and clinical data regarding tumor grade and type have been collected and were used for a comprehensive analysis.

### Blood collection and processing

For both PoVB and PB respectively, an average of 6.66 mL and 7.5 mL of blood was collected in either a 10 mL Streck cell-free DNA or an EDTA blood collection tube. All specimen samples were processed by the CSI-Cancer within 24–48 h upon collection as previously described [14, 17, 20–22]. Red blood cells were lysed in all samples and nucleated cells were plated as a monolayer on custom glass slides (Marienfeld, Lauda, Baden-Württemberg, Germany), followed by incubation with BSA, drying, and cryopreservation at −80C. Standard operating procedures for HDSCA3.0 utilize 2 slides per test of a patient sample. The filtered sample collected for Patient 26 only produced one slide, limiting the analysis. A total of 31 slides were analyzed by HDSCA3.0 for this study.

### Blood sample staining and imaging

The Landscape immunofluorescent assay (DAPI, AlexaFluor® 488, AlexaFluor® 555, AlexaFluor® 647) was used to stain all slides [14–19]. At room temperature, slides were processed in the IntelliPATH FLXTM autostainer (Biocare Medical LLC, Irvine, CA, USA) and were stained with 2.5 μg/mL of a mouse IgG1 anti-human CD31: Alexa Fluor 647 mAb (clone: WM59, MCA1738A647, BioRad, Hercules, California, USA; RRID:AB_322463), 100 μg/mL of a goat anti-mouse IgG monoclonal Fab fragments (115-007-003, Jackson ImmunoResearch, West Grove, Philadelphia, USA), and were permeabilized with 100% cold methanol. This was followed by an antibody cocktail including: mouse IgG1 anti-human CK 19 (clone: RCK108, GA61561-2, Dako, Carpinteria, CA, USA), rabbit IgG anti-human vimentin (Vim) (clone: D21H3, 9854BC, Cell Signaling, Danvers, MA, USA; RRID:AB_10829352), mouse anti-human CD45:Alexa Fluor® 647 (clone: F10-89-4, MCA87A647, AbD Serotec, Raleigh, NC, USA; RRID:AB_324730), and IgG1/Ig2a anti-human cytokeratins (CKs) 1, 4, 5, 6, 8, 10, 13, 18, and 19 (clones: C-11, PCK-26, CY-90, KS-1A3, M20, A53-B/A2, C2562, Sigma, St. Louis, MO, USA; RRID:AB_476839). Lastly, all slides were incubated with Alexa Fluor 555 goat anti-mouse IgG1 antibody (A-21127, Invitrogen, Carlsbad, CA, USA; RRID:AB_141596) and 4′,6-diamidino-2-phenylindole (DAPI; D1306, ThermoFisher; RRID:AB_2629482) and were mounted onto slides using a glycerol-based aqueous mounting media. An automated high-throughput fluorescence scanning microscopy was used to image slides at 100x magnification and collected 2304 frames per slide in four colors.

### Rare event detection and classification

Rare event detection employed the computational methodology termed OCULAR (Outlier Clustering Unsupervised Learning Automated Report) which identified and classifies rare events based on four fluorescent signal intensity (DAPI, Alexa Fluor488, Alexa Fluor555, Alexa Fluor647) for four protein biomarkers: cytokeratin (CK), vimentin (V) and CD45/CD31 (CD; combined in Alexa Fluor647) [14–19]. Data reduction was conducted manually to produce a curated list of rare events based on signal intensity, localization, and cellular morphology.

All events detected were classified into eight rare cell channel types based on the presence of fluorescent biomarkers as mentioned previously. Epithelial-like CTCs (epi.CTCs) were classified as DAPI and CK positive, and negative for VIM and CD45/CD31 (D | CK). Mesenchymal-like CTCs (mes.CTC) were defined as positive for DAPI, CK and VIM, and CD45/CD31 negative (D | CK | V). Other CTC categories of interest include the triple positive CTC with signal in all four channels (D | CK | V | CD), as well as DAPI, CK, and CD45/CD31 positive and VIM negative CTCs (D | CK | CD). Additional interesting cell categories include: DAPI and VIM positive (D | V), DAPI, VIM, and CD45/CD31 positive (D | V | CD), DAPI and CD45/CD31 positive (D | CD), and DAPI positive (DAPI only). Automatic quantification of white blood cells (WBC) in whole blood was used to present rare event enumerations as events/mL (Medonic M-series Hematology Analyzer, Clinical Diagnostic Solutions Inc., Fort Lauderdale, FL, USA).

### Statistical analysis

Paired blood samples were analyzed using a one-sided Wilcoxon signed-rank test to evaluate the enumeration differences between filtered and unfiltered samples as well as the enumeration profile change per rare cell channel-type. Statistical significance was defined by a *p*-value ≤ 0.05.

### Morphometric analysis

To visualize the morphometrics of the cellular events before and after filtration, a two-dimensional tSNE (t-distributed stochastic neighbor embedding) was used. The morphometrics used were area and eccentricity for the nucleus and the cytoplasm along with the median intensity of the four channels. The morphometrics of cellular events were compared between draws by a Wilcoxon rank sum test.

## Results

### Patient demographics

A total of 12 matched PoVB samples and 4 PB samples were obtained from six patients. All patients were newly diagnosed with PDAC. Patient demographics are provided in Table 1. An initial blood cell count was taken of each sample prior to blood processing. The WBC counts for unfiltered PoVB, filtered PoVB, unfiltered PB, and filtered PB were 3.7 (range 2.9–8.8; mean 4.42), 2.0 (range 0.7–3.1; mean 1.98), 6.8 (range 3.6–10.0; mean 6.8), and 2.6 (range 1.5–3.7; mean 2.6) million cells/mL, respectively. There was a significant reduction in the WBC count after filtration (*p*-value = 0.0078125).

**Table 1:** Patient demographics.

*s*	Patient24	Patient25	Patient26	Patient28	Patient 29	Patient 30
Age	69	73	56	65	70	77
Gender	Female	Female	Male	Male	Male	Female
Race	Asian	White	Black	Other	White	White
Ethnicity	Non-Hispanic	Non-Hispanic	Non-Hispanic	Non-Hispanic	Non-Hispanic	Non-Hispanic
BMI (kg/m2)	21.08	22.81	40.94	21.85	24.12	34.96
Size of Mass long axis	30	50	34	48	28	28
Size of Mass short axis	25	46	24	29	20	26
Location of Mass	Head	Head	Uncinate	Head	Head	Head
PoV involvement	No	No	No	No	Abutting	No
SMV involvement	No	Encasement or invasion	Abutting	No	No	Abutting
SMA involvement	No	Encasement or invasion	No	No	No	Abutting
Celiac plexus involvement	No	Encasement or invasion	No	No	Encasement or invasion	No
Abdominal LN seeding	No	No	No	No	No	No
Distant metastases	No	Yes	Yes	Yes	No	No
*T* stage	2	4	2	4	4	4
*N* stage	0	0	0	0	1	0
*M* stage	0	1	1	1	0	0
Clinical stage	1b	4	4	4	3	3
Differentiation	Poorly	Moderately	Moderately	Moderately	Moderately	Moderate-poorly

Size of mass long axis and short axis, location of mass, PV involvement, SMV involvement, SMA involvement, celiac plexus involvement, and abdominal LN seeding were determined by CT. For involvement “No” represents no or patent or unremarkable.

*PoV* portal vein, *SMV* superior mesenteric vein, *SMA* superior mesenteric artery, *LN* lymph node.

### Rare event identification and enumeration in unfiltered and filter cohort

**Fig. 2:**
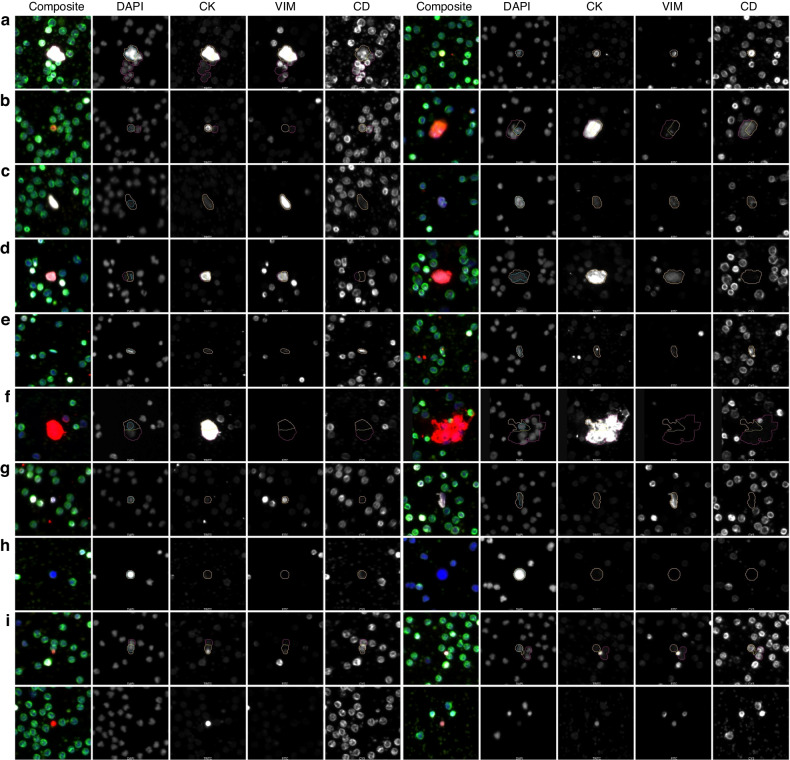
Representative Gallery of Rare Events detected across samples. **a** D | CK | V | CD, **b** D | CK | CD, **c** D | V | CD, **d** mes.CTC, **e** D | CD, **f** epi.CTC, **g** D | V, **h** DAPI only, **i** oncosomes proximal to cells (top) and standalone (bottom). Images taken at 100x magnification.

All rare cellular events detected were classified into eight rare cell channel types based on biomarker expression as mentioned prior. A representative gallery of rare events is shown in Fig. 2, showing the heterogeneity in the rare cell population within the liquid biopsies of PDAC patients.

**Fig. 3:**
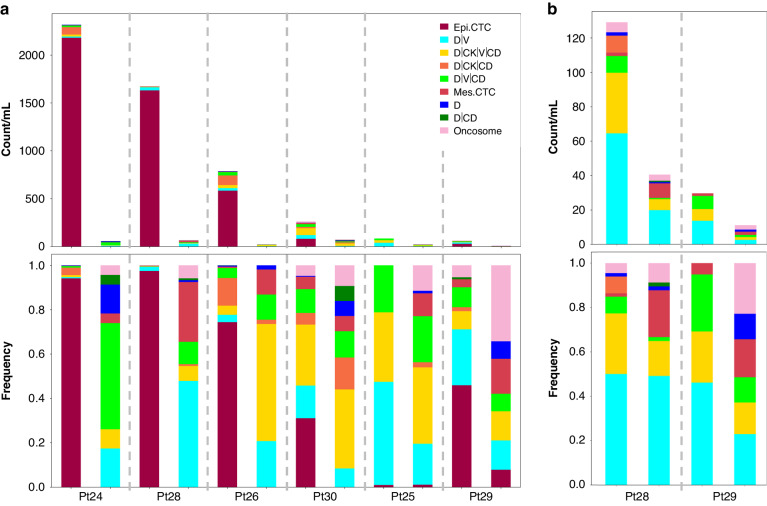
Rare event enumeration and frequency for paired unfiltered (left) and filtered (right) PoVB **a** and peripheral blood **b** samples. Enumeration (top) and frequency (bottom) plots of rare events detected by HDSCA3.0. Specific channel-type classifications are shown within each bar as indicated by the color code.

**Fig. 4:**
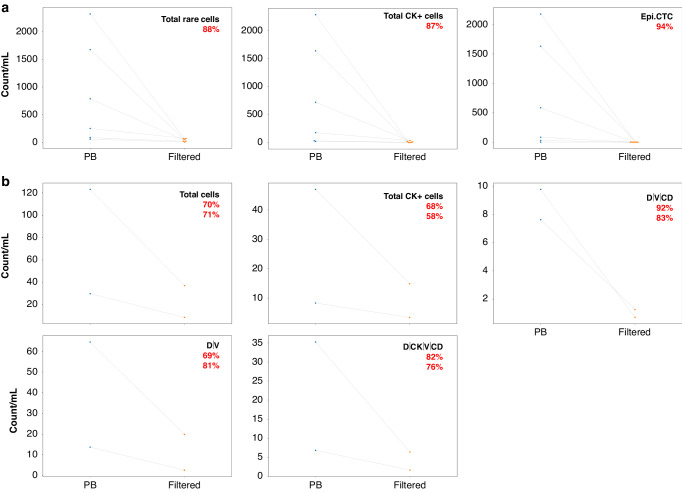
Change in cellular kinetics due to filtration in **a** PoVB and **b** PB samples. In PoVB the average percent reduction across patients is presented in red text. In PB, the red text indicates the percent reduction in Patient 28 (top) and Patient 29 (bottom).

There was a decrease in total rare cells after filtration of the blood (Fig. 3). In PoVB the total rare events, total CK+ cells, and epi.CTCs all showed a significant reduction in incidence due to filtration across all 6 patients (*p*-values = 0.03125; Fig. 4a). The total rare cells detected in the unfiltered PoVB samples had a median of 524.59 (range 62.37–2316.97; mean 864.69) events/mL while the filtered PoVB samples had a median of 42.20 (range 9.37–75.69; mean 43.32). The total CK+ events found in unfiltered PoVB samples had a median of 451.41 (range 27.28–2281.45; mean 841.70) events/mL, and the filtered PoVB samples had a median of 13.16 (range 3.43–43.05; mean 17.36) events/mL. Most samples exhibited an observable decrease in epi.CTCs after the filtration process. Patient 25 was the only patient that presented with < 1.0 epi.CTC/mL. Unfiltered PoVB samples had a median number of 333.28 (range 0.84–2180.08; mean 751.37) epi.CTCs/mL and filtered PoVB had a median of 0 (range 0–0.73; mean 0.17) epi.CTCs/mL showing a reduction on average by 94% (range 67–100% reduction).

The filtered PB also showed a decrease in the total rare events, total rare cells, total CK+ events, D | CK | V | CD, D | V | CD, D | V counts/mL due to filtration in both patients (Fig. 4b). The total rare events found in unfiltered PB samples had a median of 79.33 (range 29.68–128.99; mean 79.33) events/mL while the filtered PB samples had a median of 25.77 (range 11.11–40.47; mean 25.77) events/mL. Enumeration of total rare cells in unfiltered PB samples had a median of 76.40 (range 29.68–123.12; mean 76.40) cells/mL with filtered PB samples having a median of 22.73 (range 8.58–36.88; mean 22.73) cells/mL. The total CK+ events in unfiltered and filtered PB samples respectively had a median of 27.64 (range 8.33–46.95; mean 27.64) events/mL and a median of 9.20 (range 3.51–14.89; mean 9.20) events/mL. Enumeration of D | CK | V | CD events in unfiltered PB samples had a median of 21.01 (range 6.81–35.22; mean 21.01) events/mL and filtered PB samples had a median of 4.00 (range 1.64–6.37; mean 4.00) events/mL. Total D | V | CD events in unfiltered PB samples had a median of 8.70 (range 7.63–9.77; mean 8.70) events/mL, and filtered PB samples had a median of 1.00 (range 0.72–1.28; mean 1.00) events/mL. Total D | V cells in unfiltered PB had a median of 39.09 (range 13.71–64.46; mean 39.09) events/mL and filtered PB samples had a median of 11.21 (range 2.56–19.85; mean 11.21) events/mL.

There were two patients with matched PB and PoVB samples. The PoVB had a higher incidence of rare events than the PB. The PoVB samples presented with epi.CTCs while the PB did not.

### Rare cell morphometric analysis

**Fig. 5:**
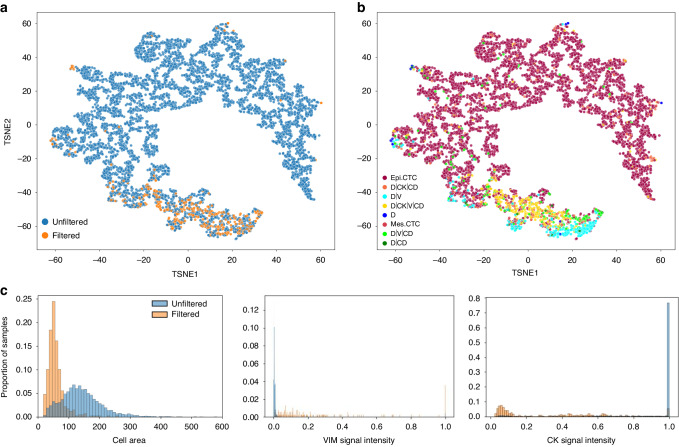
Morphometric analysis of individual events detected by HDSCA3.0 in PoVB samples before and after filtration. Depicted are tSNE plots of rare cellular events depicting the underlying morphological heterogeneity by **a** sample type unfiltered or filtered and **b** channel-type classification. Each point represents a single cell and is color-coded according to the sample type. **c** Visualization of the probability distributions for cell area, VIM signal intensity, and CK signal intensity.

Cellular morphometrics were used to further investigate the type of cells that were removed from the filtration process. A dimensionality reduction algorithm was applied to visualize the cellular heterogeneity before and after filtration in a two-dimensional plane using the median intensity for the four channels, as well as the cellular and nuclear area and eccentricity. The tSNE depicted in Fig. 5a is color coded according to sample type (before and after filtration) and shows the reduction of cells after filtration. Figure 5b shows the same cellular population color coded by classification, revealing that filtration eliminated the epi.CTCs. Taken together, Fig. 5a, b illustrate that the majority of cells remaining after filtration have neighboring morphological profiles, and with distinct profiles from the epi.CTCs. The three most significant morphometric features that best differentiate the rare cells detected before and after filtration were cell area, CK signal, and VIM signal which are represented by the probability distribution plots in Fig. 5c (*p*-value = 6.5653e-155, 1.4613e-162, 2.6776e-184, respectively). The distribution of the morphometric values for each channel-type rare cell classification is provided in Supplementary Fig. 2. The PoVB filtered circulating rare cell profile had a smaller cell area, less CK expression, and more VIM expression compared to the unfiltered paired sample.

## Discussion

This study demonstrates the initial proof of concept that blood filtration by Seraph100 can remove circulating cancer cells and may be used as a potential therapeutic device for PDAC patient care. CTCs come from the primary or metastatic lesions and circulate throughout the body in the vascular system, playing an important role in metastasis formation. Therefore, removal of these cells may be an effective treatment option. A clinical trial is warranted to prove the clinical utility of this device for therapeutic filtration to selectively remove or manipulate CTCs to treat cancer, either as primary therapy or adjunct to conventional therapy [23–25]. The ExThera Seraph 100 filter has been shown to be safe and effective for pathogen adsorption therapy for effective removal of bacteria, viruses, and fungi. In the United States it is available under Emergency Use Authorization for the treatment of COVID-19 [26–33]. The Seraph 100 filter removes pathogens from the blood by exploiting their interaction with heparin/heparan sulfate by using an ExThera adsorption media of ultra-high molecular weight polyethylene beads surface modified with covalently bonded heparin [32]. Soluble heparin is an anticoagulant drug found in mast cells and discovered by McClean in 1916 [34]. When chemically modified and immobilized on a substrate by so-called end-point attachment, it mimics heparan sulfate, which is normally expressed on the surface of the cells to mediate cellular processes such as cytokine signaling, adhesion, proliferation, and inflammation [35, 36]. Membrane-bound heparan sulfate proteoglycans include CD44, syndecans-1–4, betaglycan, glypicans 1–6, and neuropilin-1. Heparin may differentially influence cell adhesion via selectins, integrins, and neural cell adhesion molecule 1 (NCAM1, CD56) [37–39]. This may explain the selective filtration of CTCs from the blood of PDAC patients.

Therapeutic apheresis provides an important option to manage specific complications associated with malignancy and metastasis. The most commonly used apheresis modalities in cancer patient care include thrombocytapheresis, leukocytapheresis, erythrocytapheresis, therapeutic plasma exchange, and extracorporeal photopheresis [23]. Therapeutic apheresis has been associated with an overall low incidence of adverse effects (5–12%) [24]. In this small study, we observed a significant change in the WBC count after filtration of the blood. This may be indicative of a potential risk of leukopenia in clinical implementation which is common with radiation and chemotherapy treatments [25]. Clinical trials are needed to determine the safety of the device in specific clinical settings.

Beyond the CTCs, there were observable changes in the other rare cell classifications. Due to the low sample size, statistical analysis is not possible and limits the implications of these changes. Compared to the epi.CTC change that was observed, the change in the other rare cells is relatively minor. The lower incidence of detection may be indicative of the biological heterogenity of cells in a classification or “noise” due to other varables such as other pathologies (i.e. CECs in myocardial infarction [40, 41]) or population diversity. The range of rare events detected in a non-disease control cohort is 4.39–132.94 events/mL [42] which provides a sense of what the “noise” might be in a non-cancer patient sample. In this study, we present the rare cells beyond the CTC as channel-type specific classifications that indicate the expression profile given the biomarkers used (D, CK, V, CD) but these do not indicate functional cell types. The CTC classifications are hypothesized to be homogeneous populations of cells with similar biological functions. However, we find other rare cell classifications (i.e. D | CK | V | CD, D | V | CD, etc) that are heterogeneous populations which include a variety of cell types including circulating endothelial cells, fibroblasts, and megakaryocytes [16, 18, 41] as well as other rare CTC subsets [14].

In the patient cohort presented here, there were very few oncosomes detected before and after filtration. A previous study utilizing the same liquid biopsy technology showed the presence of oncosomes in the PB and PoVB in patients diagnosed with localized PDAC [42]. Oncosomes have been characterized previously in a variety of cancer types and appear to be cancer-specific as they contain oncogenic material [16–19, 43, 44]. In breast cancer, we have found that oncosomes are circulating at a higher incidence in localized disease than in metastatic disease [17], suggesting that these vesicles may be shed earlier in the disease state for some cancers. Only 1 patient in this study had localized disease limiting the analysis of stage specific correlations. Future studies in larger PDAC patient cohorts will allow for such analysis of the rare events detectable in the liquid biopsy.

This study included 2 paired PB and PoVB samples allowing for a preliminary understanding of the differences in circulating analytes between anatomical compartments. The PoVB had a higher incidence of rare events than the PB and presented with a wide range of epi.CTCs while the PB did not. In a previous liquid biopsy study analyzing samples collected from patients with localized PDAC undergoing surgical resection, the PoVB also contained a higher frequency of analytes than in the PB [42]. PoVB has been shown to have elevated CTC counts compared to PB [45] that correlate with higher metastasis rates [2, 46, 47]. Together this suggests that the peripheral circulation represents a different rare event profile, likely a subset of that observed in the PoVB. We hypothesize that the liver filters out rare events from the PoVB prior to entering the systemic circulation. The implications of this observation on the application of blood filtration are unknown. As PoVB filtration is not clinically possible, there is a concern of the efficacy of implementation in the peripheral circulation. This study only tested a fraction of the blood volume (8 mL). Clinical implementation will likely involve filtration of the whole blood volume (5 L) mulitple times.

The ability to measure the change in circulating analytes before and after treatment with the ExThera Seraph 100 filter through the liquid biopsy provides a unique opportunity to characterize the biological effect of filtration. Blood filtration with Seraph 100 filter leads to a significant reduction of CTCs. The HDSCA3.0 workflow is capable of single cell genomic and proteomic analyses [14, 15, 22, 44, 48–54] to molecularly profile the CTCs removed by the filter to further the understanding of the filter mechanism of action, as well as an opportunity to better characterize the disease in each patient.

## Conclusions

Blood purification using heparin functional beads shows promise as an efficient potential treatment strategy for PDAC patient care as a treatment adjunct or alternative. The results of this study could be relevant for other carcinomas as well. Cancer patients with CTCs treated with this therapeutic apheresis may survive significantly longer by reducing the metastatic potential of the disease.

## Supplementary information

Supplementary Information

## Data Availability

All data discussed in this manuscript are included in the main manuscript text or supplementary materials. The imaging data are available through the BloodPAC Data Commons, Accession ID “BPDC000135” (https://data.bloodpac.org/discovery/BPDC000135).
