# Supplementary material for: Determining the efficacy of ExThera Seraph100 blood filtration in patients diagnosed with pancreatic cancer through the liquid biopsy
Source: BJC Rep. 2024 Jun 27;2:47. doi: 10.1038/s44276-024-00069-3 (PMC11524105; doi:10.1038/s44276-024-00069-3)

**Supplemental Information**


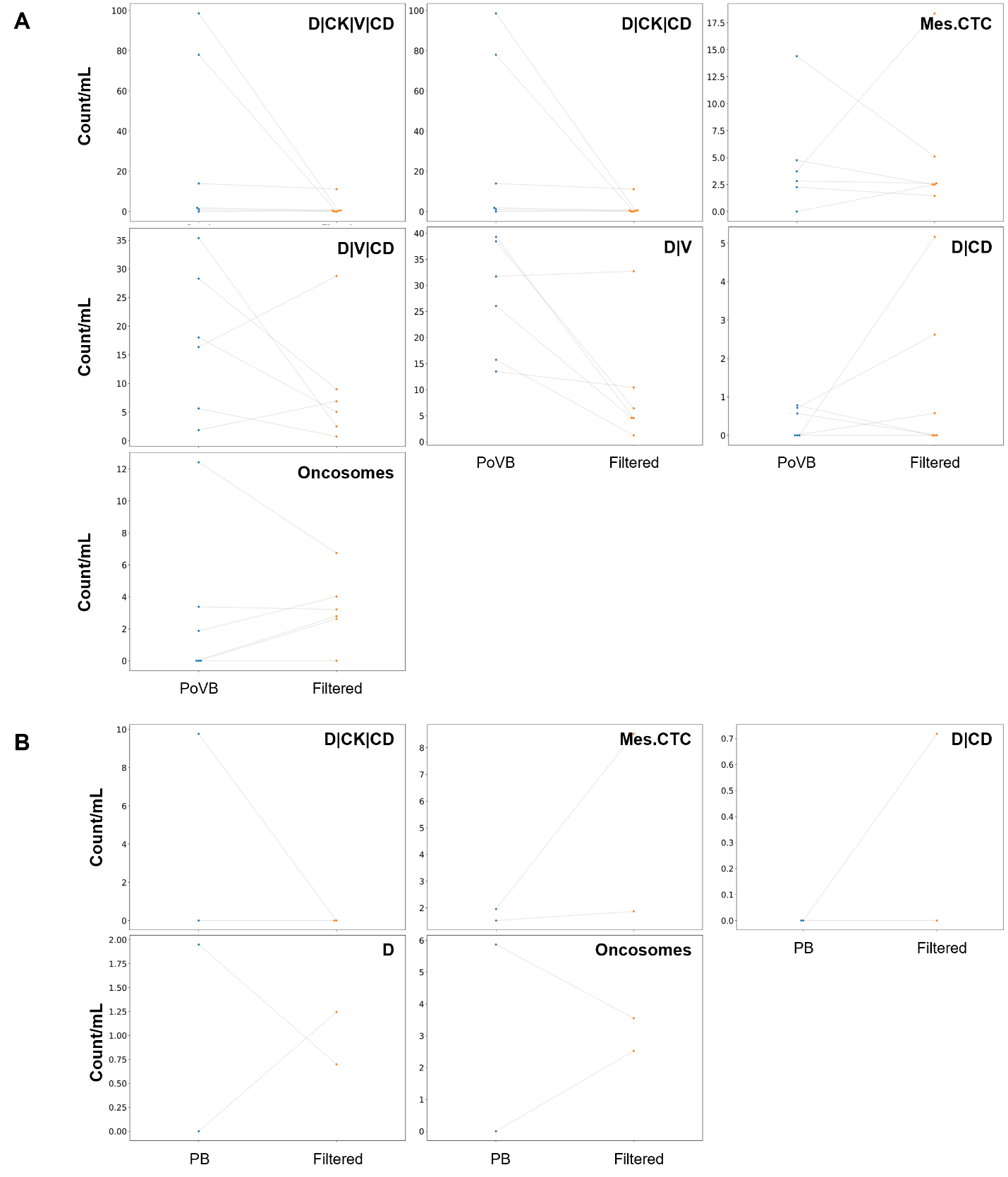


**Supplemental Figure 1.** Change in cellular kinetics due to filtration in A) PoVB and B) PB samples. In PoVB the average percent reduction across patients is presented in red text. In PB, the red text indicates the percent reduction in (top) Patient 28 and (bottom) Patient 29.

**Supplemental Table 1.** Percent change in rare event classification by patient and sample between unfiltered and filtered matched samples. Red: Decrease and Blue: Increase in the rare event after filtration.

| Patient # | Sample | Total events | Total cells | Total CK+ cells | D\|CK | D\|V | D\|CK\|V\|CD | D\|CK\|CD | D\|V\|CD | D\|CK\|V | D | D\|CD | Total oncosomes |
| --- | --- | --- | --- | --- | --- | --- | --- | --- | --- | --- | --- | --- | --- |
| 24 | PoVB | -97.41 | -97.52 | -99.66 | -100.00 | -22.75 | -74.65 | -100.00 | 75.67 | -7.20 | 57.65 | 266.13 | 262.08 |
| 25 | PoVB | -71.23 | -74.53 | -57.22 | -66.89 | -88.47 | -68.47 | 55.35 | -71.93 | 250.24 | 28.82 | 0.00 | 279.07 |
| 26 | PoVB | -97.18 | -97.18 | -97.97 | -100.00 | -82.32 | -63.78 | -99.58 | -92.90 | -47.21 | -89.41 | -100.00 | 0.00 |
| 28 | PoVB | -95.92 | -96.16 | -98.56 | -100.00 | 3.13 | 147.10 | -69.17 | 270.64 | 392.24 | 57.47 | 57.47 | 114.82 |
| 28 | PB | -68.66 | -70.05 | -68.29 | -39.48 | -69.20 | -81.92 | -92.65 | 334.81 | -100.00 | -64.15 | 71.79 | -39.48 |
| 29 | PoVB | -84.98 | -89.56 | -90.76 | -97.46 | -92.12 | -75.37 | -100.00 | -86.85 | -35.41 | 75.39 | -100.00 | -5.09 |
| 29 | PB | -62.57 | -71.07 | -57.92 | -252.34 | -81.37 | -75.94 | -83.25 | 22.45 | 0.00 | 124.55 | 0.00 | 252.34 |
| 30 | PoVB | -71.16 | -72.42 | -76.37 | -100.00 | -83.29 | -62.90 | -20.11 | -68.16 | -64.61 | 392.89 | 516.68 | -45.72 |

**Supplemental Figure 2.** Logarithmic box and whisker plots of morphometric parameters for each channel-type rare cell classification identified in the PoVB. Center line represents median, box limits represent upper and lower quartiles, whiskers represent 1.5x interquartile range, and points represent outliers. A) Cell area, B) mean VIM signal intensity, C) mean CK signal intensity.


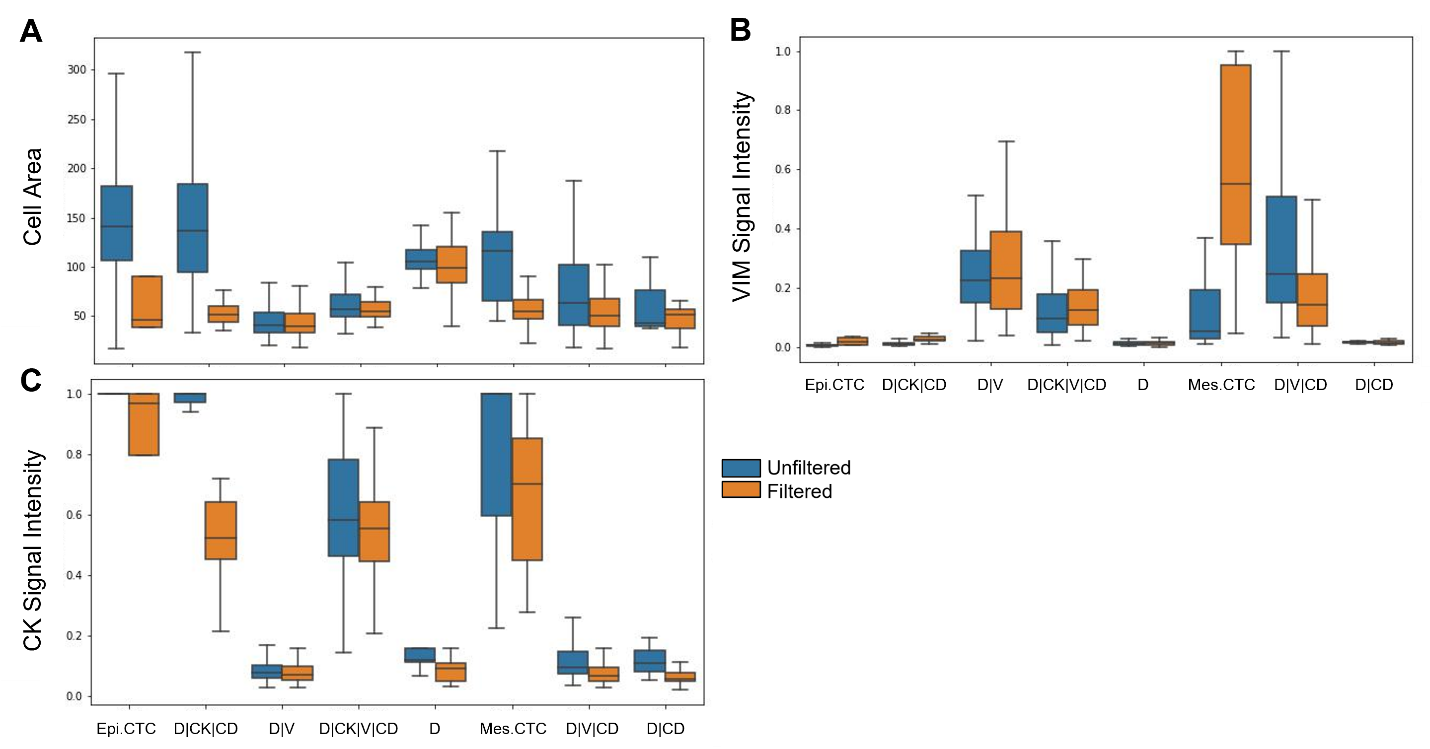

Supplement: Supplementary file 1 — Supplementary Information [file 44276_2024_69_MOESM1_ESM.docx]
